# Supplementary material for: Effects of excess sugars and lipids on the growth and development of Caenorhabditis elegans
Source: Genes Nutr. 2020 Jan 29;15:1. doi: 10.1186/s12263-020-0659-1 (PMC6988283; doi:10.1186/s12263-020-0659-1)
Supplement: Supplementary file 1 — Additional file 1: Figure S1. Body length of nematodes in the control group and sucrose (400 mM), stearic acid(500 μg/mL), sucrose and stearic acid co-treated groups (400 mM -500 μg/mL) on 6th day. Figure S2. FPKM value of different genes related to fat storage and exacerbates β-oxidations of fatty acids. Values without common letter are significantly different at p < 0.05. Figure S3. FPKM value of different genes related to fatty acids synthesized. Values without common letter are significantly different at p < 0.05. FPKM value of different genes involved in the DBL-1 signaling pathway. Values without common letter are significantly different at p < 0.05. Figure S5. FPKM value of different genes involved in the DAF-7 signaling pathway. Values without common letter are significantly different at p < 0.05. Figure S6. Immunocytochemistry images of Caspase-3 and DAPI with or without treated on 7th day. [file 12263_2020_659_MOESM1_ESM.docx]

# Supplemental Fig.1


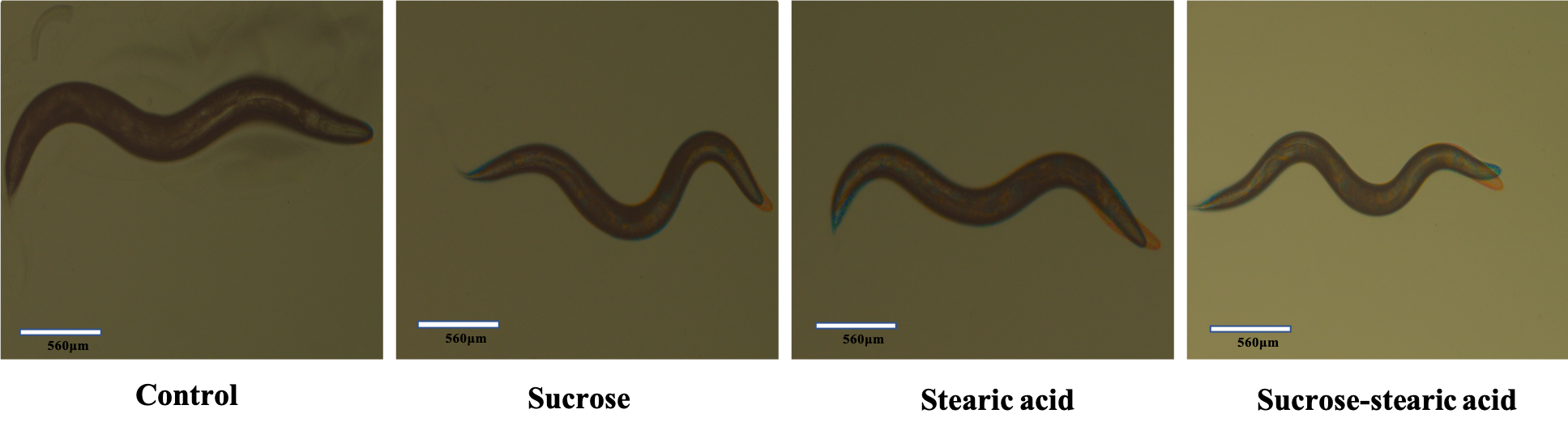


Body length of nematodes in the control group and sucrose (400mM), stearic acid(500μg/mL), sucrose and stearic acid co-treated groups (400mM -500μg/mL) on 6^th^ day.

# Supplemental Fig.2


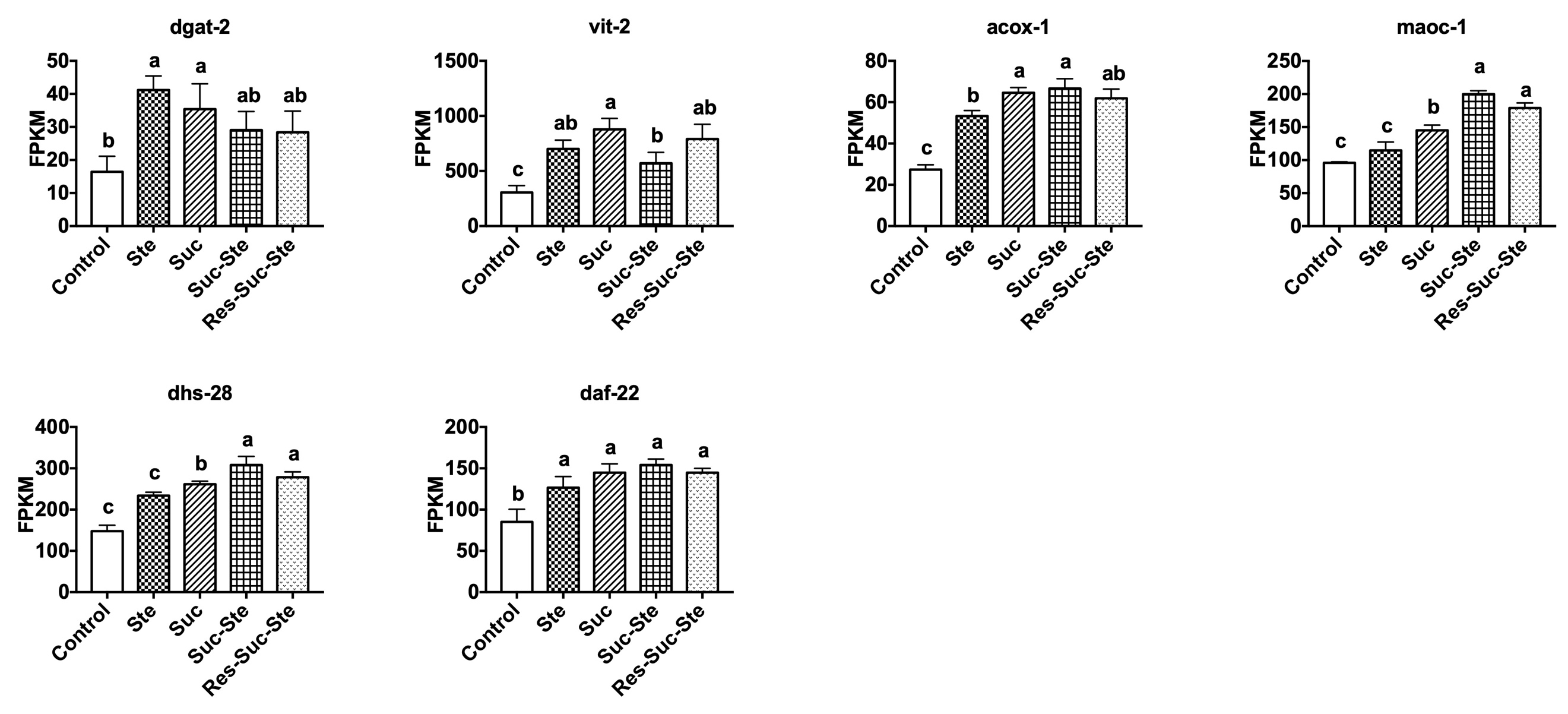
 FPKM value of different genes related to fat storage and exacerbates β-oxidations of fatty acids. Data are presented as mean ±SEM (n=3). Values without common letter are significantly different at *p*<0.05.

# Supplemental Fig.3


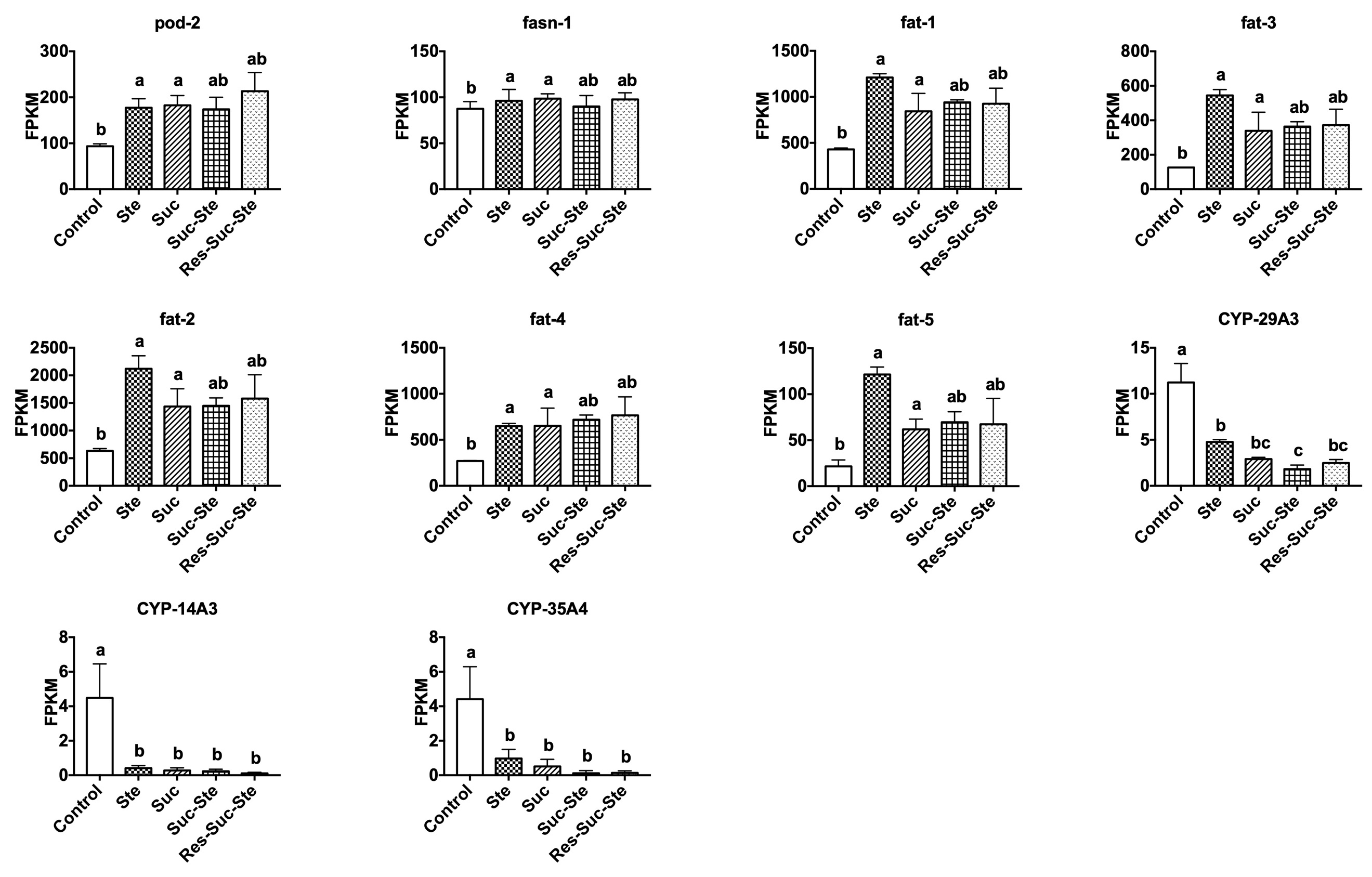
 FPKM value of different genes related to fatty acids synthesized. Data are presented as mean ±SEM (n=3). Values without common letter are significantly different at *p*<0.05.

# Supplemental Fig.4


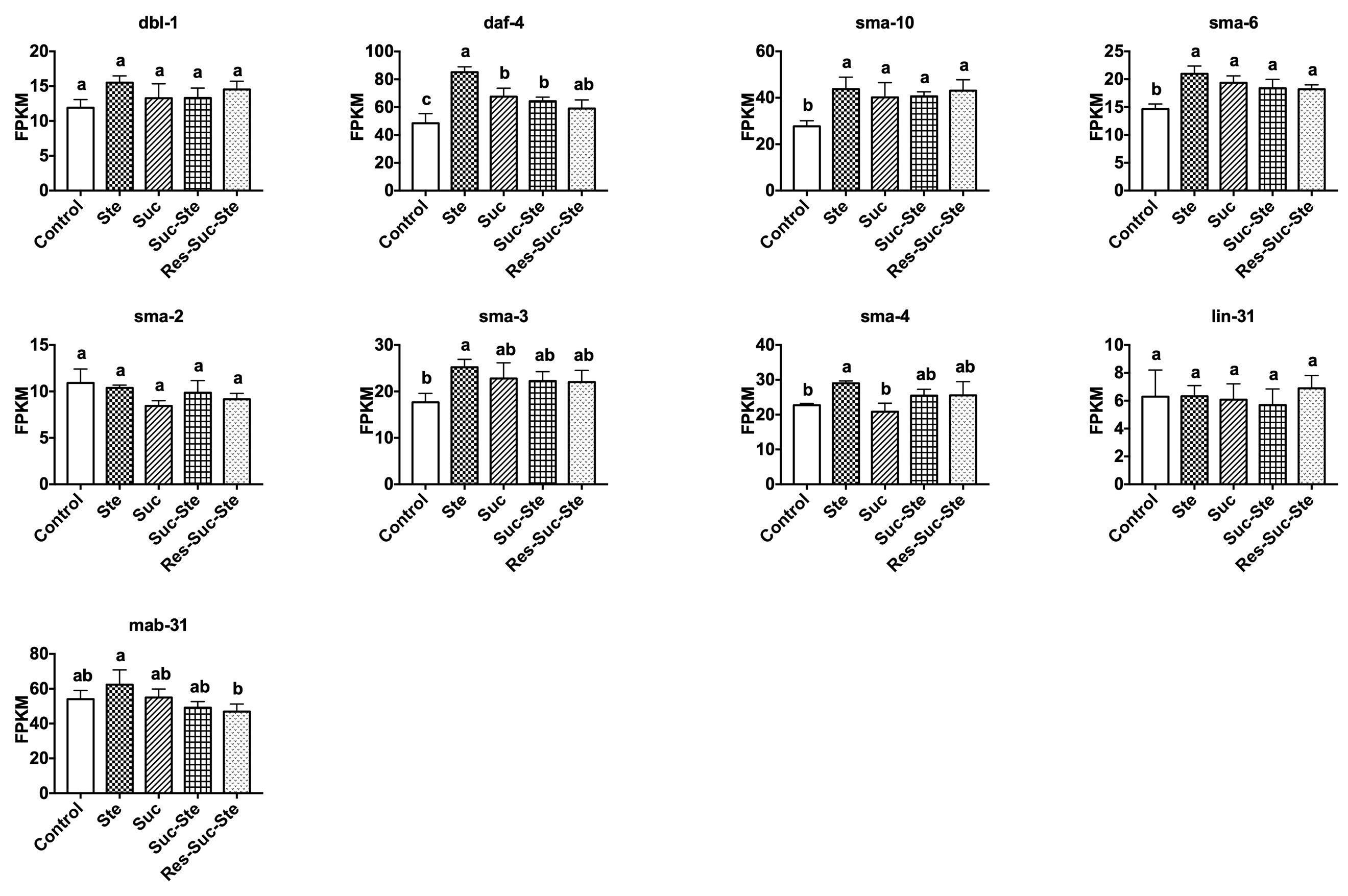
 FPKM value of different genes involved in the DBL-1 signaling pathway. Data are presented as mean ±SEM (n=3). Values without common letter are significantly different at *p*<0.05

# Supplemental Fig.5


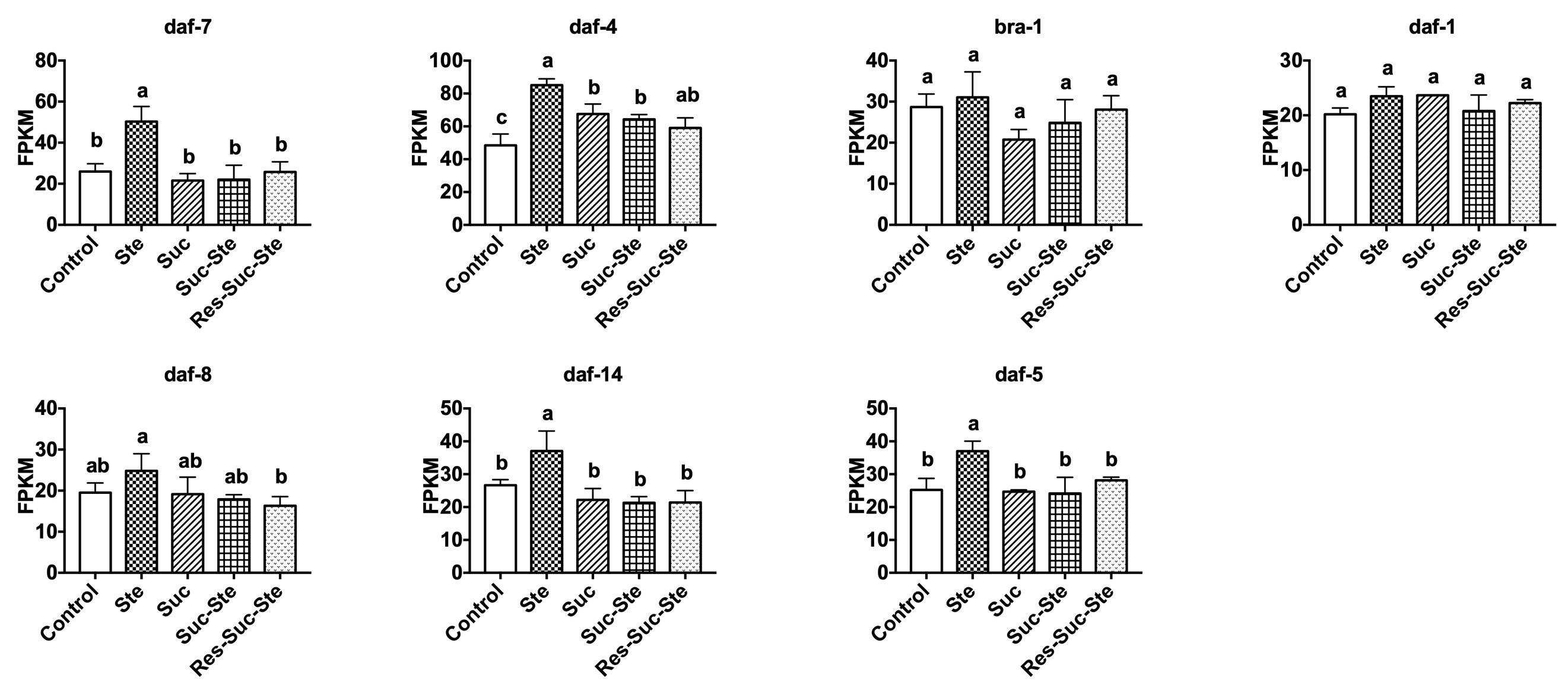
 FPKM value of different genes involved in the DAF-7 signaling pathway. Data are presented as mean ±SEM (n=3). Values without common letter are significantly different at *p*<0.05.

# Supplemental Fig.6


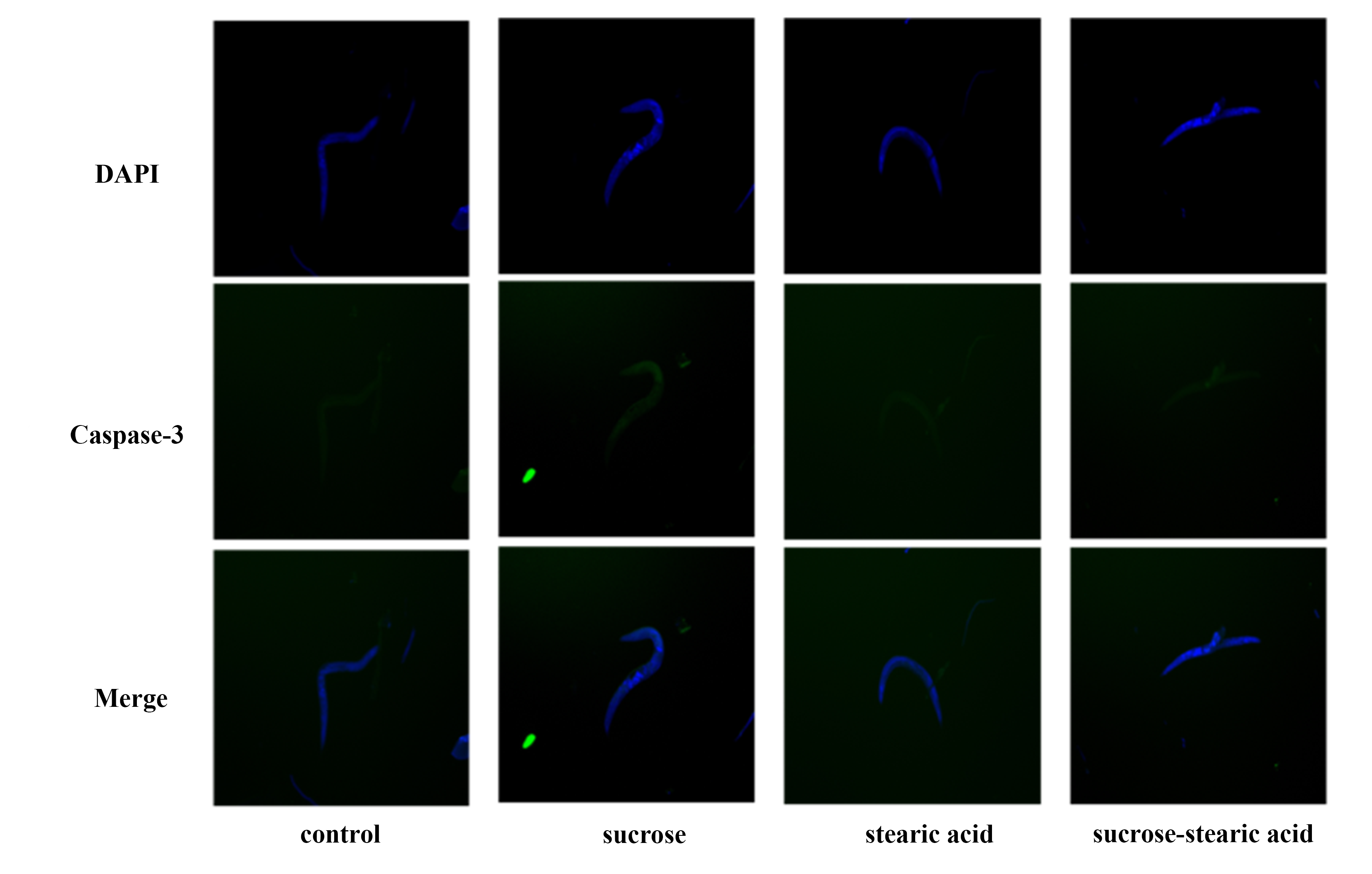


Immunofluorescence images of Caspase-3 and DAPI with or without treated on 7^th^ day. Collect sugar and lipid-treated or untreated nematodes on day 7 and fix them with paraformaldehyde for immunofluorescence assays, n=10.
